# Acquiring health resources during settlement in rural areas? Refugees' experiences of health infrastructure and leisure practices in Germany

**DOI:** 10.1016/j.jmh.2025.100381

**Published:** 2025-12-03

**Authors:** David Spenger, Stefan Kordel, Lukas Schorner

**Affiliations:** Institute of Geography, Friedrich-Alexander-Universität Erlangen-Nürnberg (FAU), Wetterkreuz 15, 91058 Erlangen Germany

**Keywords:** Health resources, Migrant agency, Migrant health, Migration, Salutogenesis, Rural areas

## Abstract

Addressing the research desideratum of health status and practices of refugees in the settlement phase as well as rural specificities in dealing with health issues, this article explores refugees’ self-reported health status as well as experiences with health infrastructure. Following a salutogenic approach to health, we consider both disease prevention and health promotion and explicitly take into account leisure practices as a way to acquire health resources. Results firstly show a persistence of self-reported diseases and manifold interactions with the settlement process, e.g. housing or language acquisition. Secondly, we found that refugees are looking for (new) leisure activities at the place of living, which allow to gain sovereignty about everyday lives and thus improve their health status. Based on our results, we suggest to take up post-medicinal and life-course approaches in future research designs for a more profound understanding of migrant health.

## Highlights

• The health status of refugees can best be understood from a lifeworld perspective.
• A salutogenic model of health considers stressors to be part of everyday life.
• Refugees find it difficult to access and navigate the healthcare system in rural areas.
• Refugees’ leisure activities are perceived to be beneficial to health resources.

## 1 Introduction

Large socio-epidemiological surveys and analyses point to an interrelationship between social conditions and health inequity (Marmot, 2015; Siegrist and Marmot, 2006; Wilkinson and Marmot, 2003; Wilkinson and Pickett, 2010). Accordingly, health status differs among populations both worldwide and within countries; at the very least, the spatial configuration of health infrastructure and ease of access to them have an impact on individuals’ health status. Migrants, vulnerable and marginalised populations, including individuals facing precarious living conditions (homeless people, or those with insecure status like humanitarian immigrants), have therefore been the focus of public health research and interventions (e.g. Hawkes et al., 2021; Vasey and Manderson, 2012; WHO, 2019). With regard to migrants, existing databases make it difficult to conduct elaborate analyses, as the category of ‘migrant’ is not used uniformly (Hövener and Wieler 2023, for Germany). The field of health has often had a shadowy existence in migration research, and has only gained special attention in recent years (in the German context, see for example SVR, 2022). Most studies that address migrant or refugee health in Europe have focused on what happens in transient spaces, such as camps or shelters along the route, or in the places where they first arrive, such as the island of Lampedusa (Pasta et al., 2019), countries they pass through, such as Türkiye (Şafak-Ayvazoğlu et al., 2021), or on conditions shortly after their arrival in their (provisional) destination countries (Cinaroglu, 2020). What happens after this – during the period of settlement, when refugees start to become acquainted with a place, work their way through local structures and are entitled to access regular health structures despite having particular needs, has not been at the core of scientific debates so far. However, because individual health status and access to health facilities interact with social inclusion, and ultimately have an impact on the integration process (Ager and Strang, 2008), we need to improve our knowledge of refugee health in the settlement phase. In this article, ‘settlement’ refers to the phase in which refugees have already been granted humanitarian protection and have thus gained the autonomy to choose their place of residence. Simultaneously, we understand settlement as an ongoing and dynamic process of negotiation about staying or leaving (cf. van Liempt and Bygnes, 2023), related to the question of whether individuals or family members can live a ‘good life’ and realise life goals. Regarding the latter, health and well-being is of crucial importance.

In the European Union, from 2015 onwards, Germany became the member state hosting the highest absolute number of humanitarian immigrants. Like some other European countries, Germany uses a dispersal scheme to distribute asylum seekers and refugees all over the country, which explains why they also arrive in rural areas (Weidinger, 2021). In terms of health infrastructure, German rural areas are well served by general practitioners, not least as a result of recent political interventions, yet those areas are characterised by a shortage of specialists and sometimes also of clinics close to where people live (Neumeier, 2018). Following a salutogenic approach, which focuses on factors beneficial or damaging to health, we aim to widen the lens, considering leisure practices as a means to acquire health resources and improve the way one deals with the (potential) burden of disease. Plenty of studies show the impact of leisure activities for improving both mental health status (Earl et al., 2015; Fenton et al., 2017; Lackey et al., 2021) and physical condition (for example, Iwasaki et al., 2014). Other studies indicate that leisure activities in a neighbourhood play an important role in supporting social networks and bonds, improving social cohesion (Lewis, 2015) and therefore strengthening a sense of well-being (Correa-Velez et al., 2010). In the settlement phase, refugees regain power to decide how to spend their (free) time. Besides, rural areas are usually well equipped with natural assets such as green and blue spaces, whose positive effects on health have often been cited (for example, Claßen and Bunz, 2018; Hartig et al., 2014) and which also offer opportunities for informal social exchange (Neal et al. 2015), and encourage physical activity (Romagosa et al., 2015). In this respect, rural areas have the potential for developing health resources, especially for vulnerable groups such as refugees. Existing studies, however, tend to focus predominantly on urban areas and a deliberately rural perspective is often missing.

Based on these preliminary considerations, the aims of this paper are,

(1) to identify refugees’ self-reported diseases and their interrelationship with other realms of everyday life in the settlement phase,
(2) to provide an overview of refugees’ experiences of health care infrastructure, actors and practices, and
(3) to highlight refugees' leisure practices as health resources in rural areas.

The remainder of this article is structured as follows. In Section 2, based on Antonovsky’s salutogenic understanding of health (1996), we outline the research context of refugee health, their experiences of health care infrastructure and leisure practices and provide a brief introduction to rural specificities in Germany. In Section 3, we present our data and methods, while Section 4 describes our results and responses to the research questions above. In Section 5, we discuss our findings, while Section 6 contains the conclusion and outlook for further research.

## 2 Research context

### 2.1 A salutogenic approach to health

Following a salutogenic approach, we highlight interactions between individual health and place and thereby go beyond health-related infrastructure that focus on treating acute diseases. The salutogenic model goes back to the medical sociologist Aaron Antonovsky (1996), who understands health not as a dichotomy between health and sickness, but as a dynamic process between the poles of health and disease. Throughout their lives, individuals are constantly surrounded by factors harmful to their health (i.e., stressors) as well as factors that promote health. The way in which stressors are dealt with depends on a ‘sense of coherence’, a generalised perception of the world as comprehensible, manageable and meaningful (Antonovsky, 1996, p. 15), which is shaped by specific life experiences. Health resources help individuals to increase their sense of coherence and facilitate their ability to cope with future stressors. Referring to the determinants of health as presented by Dahlgren and Whitehead (1991), we conceptualise individuals’ health resources as the possibility of making use of material and social conditions, such as health infrastructure, green/blue spaces, leisure activities and social contacts. In the case of forced migrants (both asylum seekers and refugees), a life course approach is crucial for understanding their health resources and status (Spallek et al., 2011). First, they experience health problems *en route*. Second, during the settlement phase they are exposed to stressors like frequent residential moves, poor living conditions or a precarious financial situation, which leaves them insecure and may prolong existing illness or cause new health issues like mental disorders (Hynie, 2018). Besides, health problems may themselves impede settlement and access to other areas of integration, such as employment, housing, education and social contact, which may result in further social exclusion (Hynie, 2018; Kordel et al., 2023), ultimately reducing individuals’ sense of coherence.

### 2.2 The health situation of refugees

It is widely known that experiences of war, violence, persecution and distress both before and during flight (Tinghög et al., 2017) can cause post-traumatic stress disorder (PTSD), anxiety or depression (Bogic et al., 2015). Several studies have found that symptoms of these forms of psychological distress are highly prevalent among forced migrants in Germany and occur more frequently than in the resident population (Hajak et al., 2021). However, the proportion of refugees who suffer from these issues varies greatly between studies. For example, Georgiadou et al. (2018) found symptoms of PTSD in 11.4 % of respondents, while Comtesse and Rosner (2019) identified them in 45.4 %. While asylum seekers and people whose asylum applications have been rejected suffer more from symptoms of PTSD, recognised refugees more frequently show symptoms of depression (ibid.). Among refugees who had already received a residence permit, Borho et al. (2020) found that symptoms of psychological distress persisted over a period of 1.5 years. Refugees’ physical health problems have been less researched than their mental health. This might be due to the relatively young age and therefore better than average health of refugees in Germany (Metzing et al., 2020), which is often referred to as the ‘healthy-migrant-effect’ (Spallek et al., 2011). Data from a quantitative study among 2021 forced migrants at a reception centre in Germany show that they are less likely to suffer from chronic illnesses, but that those who reported chronic physical health problems suffer from cardiovascular diseases (25.8 %), diabetes (21.8 %) and back and joint disorders (8.9 %) (Schröder et al., 2018). In an analysis of data from an ambulatory refugee clinic in the Federal State of Saxony (*n* = 2753), Goodman et al. (2018) showed that humanitarian migrants were frequently diagnosed with respiratory diseases and infections shortly after their arrival in Germany. Injuries and mental illness were diagnosed far less frequently.

In the treatment of mental ill health, studies indicate that structured psychotherapies can be an effective means of recovery from stressful migratory experiences (Silove et al., 2017). Legal barriers mean that access to psychotherapies is unlikely to be granted during the asylum-seeking process (Frank et al., 2017). Thus, the settlement phase might be an important time for therapeutic intervention. However, serious mental disorders may remain (Fazel et al., 2005) and the duration of effectiveness of mental health interventions is related to refugees’ degree of exposure to daily stressors, as well as core psychosocial systems, which go beyond individual health status to include the health status of household members (Silove et al., 2017). Therefore, individuals’ post-migratory circumstances, and the settlement phase in particular, are assumed to be highly relevant for the mitigation of health problems. Finally, overemphasising these vulnerabilities may lead to a disregard for individual competence and foster stigmatisation (Ali-Hassan et al., 2021; Hynie, 2018). Accordingly, although a salutogenic approach to health is often absent, it has the potential to focus on individual agency and health promotion strategies in particular.

### 2.3 Refugees’ experiences with health care infrastructure and leisure practices

In terms of the treatment of both acute and chronic diseases, studies have reported the various obstacles faced by migrants and refugees in particular. Empirical studies highlight an absence of government funding for treatment costs, a shortage of doctors able to attend to refugees’ needs, or willing to provide treatment at all. Moreover, refugees feel that the language support offered during consultations is inadequate (Correa-Velez et al., 2013; Schröder et al., 2018). In addition, information about the health system in the country of arrival, for example about prescription medicines, is often sparsely provided and health services are sometimes used inappropriately (for instance when people make an emergency call for minor or routine medical needs) (Kordel et al., 2023). Moreover, differences in treatment practices can result in misunderstandings and uncertainties (Ager and Strang, 2008).

Although often overlooked in terms of migrant health, leisure is an important health resource. Studies show that leisure activities can help individuals cope with mental disorders (Gerber et al., 2021; Hurly, 2018) and strengthen social connectedness and physical health (Sampson and Gifford, 2010; Suto, 2013). Being physically active, for instance, can help reduce cardiovascular risk markers (Gerber et al., 2021). Studies indicate that during the arrival phase, asylum seekers’ physical activity depends on the social composition of the reception centre and the organisation of activities on site (Andersen et al., 2021; Haley et al., 2014; Waardenburg et al., 2019) In the settlement phase, different ideas about leisure may prevent refugees from continuing their usual activities. In their study in regional Australia, Reis et al. (2020) found that most participants had engaged in physical activity for leisure before their arrival and found it challenging to integrate into new leisure structures. A considerable number of refugees, according to that study, acquired a ‘new vocabulary of leisure’ and embraced new leisure practices, which they linked to a ‘Western’ lifestyle with its greater orientation towards a free choice of leisure opportunities, especially for women. However, it is also important to consider limitations on leisure activities. Refugees may lack control over their time due to childcare and domestic responsibilities (Hurly, 2018; Ryom et al., 2022), and a lack of money (Reis et al., 2020) can also restrict access to leisure activities. It is also worth noting that recreational activities may change depending on the season (Hurly, 2018). Moreover, refugees can also experience discrimination and racism in leisure settings (Kloeck et al., 2013, for Muslim women in the Netherlands), which discourages participation and can increase individual stress levels (Walker et al., 2011).

### 2.4 Rural specificities in Germany

Among other things, rural areas in Germany are characterised by limited accessibility of crucial health infrastructure like hospitals, doctors or pharmacists (Kriwy et al., 2020). This stems from a political decision to use outpatient and inpatient health facilities in the city to help care for patients in the surrounding area (Warth et al., 2021). Urban-rural differences can therefore be observed in the provision of general practitioners (Greß and Stegmüller, 2011; Neumeier, 2018), specialist doctors (Neumeier, 2018) and other medical service providers (van den Berg et al., 2015). Culture-sensitive medical services that can meet the specific needs of different migrant groups are less common in rural places. In terms of recreational facilities, rural areas tend to have a limited range of leisure facilities, which can hamper individuals’ physical activity and health-promoting recreational activities (Warth et al., 2021). Moreover, most rural places have green space, which offers opportunities for building up health resources, whereas rural areas are often considered to be beneficial to health per se (see Gesler, 1992 on *therapeutic landscapes*). Addressing the existing research *desideratum* in the context of disease prevention and health promotion among humanitarian migrants in rural areas, we explore refugees’ experiences of accessing medical facilities as well as their leisure practices.

## 3 Methods

This paper is embedded in a larger joint research project that focused on refugees' orientation towards staying in rural areas (2018–2021). Research was conducted in small towns and villages in eight rural districts in four German Federal States. In terms of their health-related infrastructure, the study sites had features similar to those mentioned above. While general practitioners or pharmacies were commonly available in residential areas, or at least easily accessible (Neumeier, 2018), specialist doctors like psychotherapists, or even hospitals were relatively difficult to access, not least due to a lack of public transport and individual mobility. Some of the study sites were characterised by natural amenities like large forests, lakes or rivers, including the Bavarian Forest or the Hesse Highlands, which are also designated tourism spots, although the availability of leisure facilities was not limited to those regions.

The empirical data stem from 139 qualitative interviews conducted among a total of 192 refugees. The sampling process had to consider refugees’ life-worlds and daily routines. Thus, a variety of methods was used to recruit interviewees. At first, contact was established via gatekeepers (volunteers or social workers, for example), while snowball sampling was implemented to expand the circle of participants. At the same time, some participants were recruited by making contact in relevant locations, such as integration courses or asylum cafes. Since vulnerable groups were addressed, ethical procedures were of crucial importance (Clark-Kazak, 2017). Since these differed among the four institutions involved, the study was eventually approved by the ethics board of the institution with the highest ethical standard. During a so-called ice-breaker meeting before an interview, participants were informed about the aim of the project and data protection, giving them time to think about participating, in some cases supported by interpreters, before giving their informed consent. Interviews were then conducted either in German or, if desired, with the support of an interpreter. We stopped recruiting once we had achieved data saturation in terms of both depth and breadth, a decision arrived at jointly with co-researchers from the refugee community. The interviews lasted between 60 and 235 min. Participants originated from Syria (*n* = 110), Afghanistan (*n* = 22), Iraq (*n* = 19), Eritrea (*n* = 12) and other countries (*n* = 29); their average age was 34.3 years, 42.2 % were female and 72 % lived with at least one child. The interviewees were in the settlement phase; that is, they had lived in one place in Germany for at least six months (five years at the maximum) and mostly held a recognised humanitarian protection status. They were therefore entitled to use the regular public health system. The qualitative interviews were supplemented by the visual tools of (im)mobility biography (Kieslinger et al., 2020) and mobility mapping (Weidinger et al., 2021). (Im)mobility biographies captured migrants’ places of residence and living conditions over time, while mobility mapping made it possible to record individually important places and evaluate their accessibility in everyday life. Here, the ascription of meanings to places was explicitly considered.

The analysis was carried out by combining both text and visual data. First, interviews were transcribed and coded while maintaining inter-coder reliability. Using Ager and Strang’s (2008) integration model as a conceptual framework during the coding process, codes were derived deductively and expanded inductively. Second, graphic elements were analysed in a product-oriented way (Lutz et al., 2003) and narrative analysis was applied (Kohler Riessman, 2008). The authors translated into English all quotes used in the results presented in the next section.

## 4 Results

What follows presents first of all participants’ perception of their health status in the place they are settling. Secondly, health-related practices encompassing both treatment and activities that may contribute to health are analysed from a place-based perspective.

### 4.1 Self-reported health status of refugees

Refugees described their health status in relation to time and place. Because health conditions tend to interact and persist over time, it is reasonable to structure the results according to reported health status during both the displacement and the post-migratory phases when the interviews took place. During forced migration, refugees were exposed to violence, war, persecution and poor housing conditions in camps. Accordingly, interviewed refugees reported physical health problems such as viral disease (D_VIII_REF_124) ,^1^ hepatitis (A_I_REF_011) and varicella (A_II_REF_027) and mentioned that acute diseases, including thyroid disease, had not been treated on the flight route (A_I_REF_012). Their current state of health had also been affected by experiences of torture (for example in a prison in Syria (D_VIII_REF_125)), and congested transport on the escape route, which triggered shoulder and back injuries (D_VII_REF_107). Psychosomatic indications, especially stress-related, such as hypertension (C_V_REF_072, C_VI_REF_096), hearing loss (D_VIII_REF_139) or irritable bowel syndrome (B_IV_REF_049) were also reported. In addition to acute diseases, chronic disease was also relevant, including anaemia (A_II_REF_023) or diabetes mellitus (A_I_REF_002, B_III_REF_036). Psychological illnesses mainly included trauma and its effect. Mental disorders ranged from anxiety disorders (phobias, fear of people, C_VI_REF_092), to panic attacks (C_V_REF_081, D_VII_REF_102) to depression (A_II_REF_018). The latter was also associated with suicide attempts (A_I_REF_013).

*After we moved to [SMALL TOWN A], I was sick. And when we were here [SMALL TOWN B], I got sicker. I tried to kill myself several times. I went to a psychiatrist. I was under pressure here and I was often in the hospital, because I didn’t have any contacts here in [SMALL TOWN B]. I didn’t meet anyone.*

Female, 33, Lebanese, approx. 8 years in Germany (A_I_REF_013)

Other reported psychological problems comprised nervous collapse (B_III_REF_029, 048, C_V_REF_077), sleep and concentration disorders (C_VI_REF_094, D_VII_REF_102, D_VIII_REF_129) or general weakness (C_VI_REF_094). Finally, refugees were not only concerned about their own health, but also about that of their children (A_I_REF_013), parents (C_VI_REF_101) or relatives in their country of origin (D_VIII_REF_133), which in turn could have an impact on their own mental health. In relation to children, in particular, they reported growth disorders (A_I_REF_001), vision and hearing disorders (C_VI_REF_083) or speech disorders (C_VI_REF_098).

These results confirm that health status also interacts with other dimensions of settlement. On the positive side, the authorities gave refugees with illnesses the opportunity to move into their own flat (A_I_REF_012) or assistance to move to a different flat, as for example in the case of a respondent’s daughter, who suffered from a disability and had back surgery (C_VI_REF_099). The following quote shows that people were able to move into a private dwelling even before receiving a residence permit.

*No, before, because my husband has cancer. He can’t stay in the camp because there’s a lot of bacteria in the camp. (…) It has to be clean and must not smell of anything. Yes. And he needs rest, because his illness comes from the psyche, not the body. Yeah, that’s what the doctor said. (…) A year ago, we got a flat. But it wasn’t easy. My friend is in the [NAME of political party] and she helped us. She fought with the immigration department.*

Female, 30, Syrian, approx. 5 years in Germany (A_I_REF_012)

On the other hand, chronic illnesses may prevent access to or regular participation in integration measures, while participants also reported that absence due to illness caused difficulties in school (B_IV_REF_064). In addition, health effects were reported in relation to starting work, mainly back problems that made it impossible to perform physical activities (A_II_REF_020, B_III_REF_029, B_IV_REF_059, C_VI_REF_100).

### 4.2 Refugees’ experiences with health care infrastructure

In the salutogenic approach, health-related practices encompass both the treatment of acute diseases and the promotion of health (through leisure practices, for instance). In terms of the former, the mobility mapping showed that refugees mainly sought doctors within walking distance in their residential area (139 out of 170). Doctors outside their residential area might be reached by public transport (110 out of 172) and, to a lesser extent (68 out of 172), also by car. For doctors located a long distance from a respondent’s place of residence, long travel and waiting times meant that a doctor’s visit took all day. To access doctors, respondents got a neighbour to give them a lift (C_V_REF_069) or took their children with them for company. Refugees preferred doctors they could talk to in their native language but these were often located in larger cities.

*[speaking Arabic] Actually, if we want to go to a doctor, we go to a family doctor. There is a clinic, we go there if we have a cold or the flu. [speaking English] For example: If I want to, I go to a paediatrician. I need three hours. You need an hour by train to get there/ It’s easier here (at the place where he lives).*

Male, 33, Syrian, approx. 3 years in Germany (B_III_REF_044)

Therapies included medication (C_VI_REF_086) and frequent hospital stays (A_II_REF_020, D_VII_REF_109, 116). Refugees reported culturally-induced uncertainties about patient-doctor interaction and pointed to undifferentiated therapies (B_IV_REF_052, D_VII_REF_114). A general practitioner’s suggestion to drink water was not considered as an appropriate therapy, and it was unfamiliar to some that doctors might exercise restraint when prescribing medication.

*Actually, there is something [laughs] that surprised me. Here, if you want to see a doctor, you have to make an appointment. And if you have an appointment, you have to wait four or five hours. And even if you go in (. . .) [laughs], you don’t realise that this man is a doctor [laughs] and helps you get well. In our home country, when you go to a doctor, he gives you a big bag of medicine [laughs].*

Male, 51, Syrian, approx. 3.5 years in Germany (C_VI_REF_083)

As the above quote indicates, arranging appointments may be unusual from the perspective of many refugees. They did not understand why doctors repeatedly prescribe analgesics and focus only on symptoms rather than trying to find causes (B_IV_REF_052, D_VII_REF_114). They might also fail to take the correct dose due to a lack of explanation and communication deficits (C_V_REF_070). After all, seeking a doctor was associated with experiencing discrimination, which could aggravate an individual state of health (C_VI_REF_083). While some people told us about interventions and therapies for both acute and chronic diseases, many families also reflected on experiences in hospital during the birth of a child (e.g. B_III_REF_033, 043).

### 4.3 Refugees’ experiences with leisure practices

Above, we have focused on refugees’ medical treatment. We now go on to emphasise leisure practices. Leisure time is commonly understood as periods in everyday life when one can freely dispose of one’s own time and which have an effect on self-determination. Leisure activities mostly took place in the municipality of residence (266 out of 392). Respondents mostly got there on foot (206 out of 266) or, to a lesser extent, by bicycle (35 out of 266). Public and accessible spaces played an important role and, as the following quote stresses, fulfilled several functions, from practising sports to meeting people. Easy access and personal safety were also highlighted:

*When I have free time, I always go to the spa gardens here, it's very nice. You can go for a walk, play soccer. Meet a few people. I always go there when I have time to go for a walk. I can also go there when I'm alone. It's quite nice.*

Male, Eritrean, 20–30 years old, approx. 3.5 years in Germany (C_V_REF_081)

Places for leisure activities further from home were mostly reached by public transport (70 out of 126) or car (31 out of 126) or sometimes by getting a lift with friends or other social contacts (17 out of 126). With regard to sporting activities, refugees mainly mentioned regular visits to the gym (A_I_REF_016, B_III_REF_042, 046, B_IV_REF_062, D_VIII_REF_124, 125), volleyball, basketball and soccer training, either in a club (D_VII_REF_103, D_VIII_REF_123) or independently (B_III_REF_045, D_VIII_REF_124), pool swimming (C_V_REF_070, D_VIII_REF_125) and occasionally boxing training, table tennis, badminton, cycling, judo or chess (A_I_REF_005, 011, A_II_REF_017, 020, D_VIII_REF_132). Some refugees also attended cultural events such as the theatre or cinema (D_VIII_REF_125), although these tended to be held in larger cities. Others also valued the fact that they could spend their leisure time at home reading, playing video games, listening to music or watching television (B_IV_REF_050, D_VII_REF_104).

To a great extent, respondents valued their leisure activities, and associated them with direct positive health effects. They spoke of walks in the park and going into the countryside (A_I_REF_009, B_III_REF_030, 034, C_VI_REF_101) or meeting friends at the fair (D_VIII_REF_124) as stress relief. Doing sports and singing were seen as a positive contrast to problems in the country of origin and as generally beneficial to health (A_I_REF_011).

*IP [speaking Farsi]: But I especially look forward to Friday, when I meet up with my friends for [prayer group]. Or also to Saturday, to running, or on Monday, to doing sports.*

*I: That's especially nice for you, because then you do something for yourself there […]?*

*IP [speaking Farsi]: Fridays are nice because I meet with the others and on Saturdays and Mondays I do something for my health.*

Female, 27, Afghan, approx. 3,5 years in Germany (C_VI_REF_085)

Respondents also indicated that activities involving social contact reduced their stress and feelings of loneliness (D_VII_REF_103, 104, D_VIII_REF_124). Similarly, meetings or picnics with the local population in leisure time were seen as positive (A_II_REF_023), while joint activities with their families were also highly valued (A_I_REF_005, C_V_REF_080).

Proximity to natural amenities such as lakes, rivers or forests (A_I_REF_001, 013, A_II_REF_020, 023, B_III_REF_048, D_VIII_REF_122, 138, 139) was considered to be beneficial to health and as stress-reducing (C_V_REF_070, C_VI_REF_094). Accordingly, many participants reported joint activities such as cycling or walking with family and friends. Refugees also attributed positive effects to independent gardening (C_VI_REF_093) as well as to spending time in allotment gardens (A_II_REF_020, 27).

*[speaking Farsi]: […] It’s relatively quiet. We have a garden. […] Once a week, we have a barbecue in the garden with the family. We planted some flowers and some herbs there. It’s fun to take care of the garden. Half the day I go to school here. (…) So sometimes I sleep for one, two hours, or I’m busy. If I have the nerve, I’ll learn what I’ve been taught again.[…] And if I can, I drive to [SMALL TOWN] to someone I know so I don’t feel so lonely.*

Male, 40, Afghan, approx. 3 years in Germany (C_VI_REF_093)

Refugees also experienced some leisure places as inaccessible. Because of the shortage and lack of diversity of recreational facilities in rural areas, they had to give up some of the leisure habits and everyday rhythms they had done in their country of origin (C_VI_REF_092, D_VII_REF_102). The lack of facilities in rural places was evaluated relationally and often compared with opportunities in other places in Germany where they had previously lived (B_III_REF_042). They were also critical of the lack of transport, and the large amount of time and money involved in accessing the leisure facilities they wanted to go to (A_II_REF_017, C_V_REF_076). Exclusion was also evident, as certain leisure practices provoked social resistance or were associated with perceived annoyance (B_III_REF_039, C_VI_REF_093). Women, especially, also reported discrimination, because they were not wanted in certain sports, such as volleyball, due to religious dress practices (C_V_REF_073). Refugees might also self-exclude, as for example in the case of swimming pools (D_VII_REF_109). Fraught or difficult social contact, especially with the local population, could result in additional stress (as reported by D_VII_REF_120 for example) and feelings of loneliness with further implications on health, e.g. one's alcohol consumption practices (D_VIII_REF_125).

At the same time, refugees learnt about and appreciated new leisure activities (C_V_REF_071). Women in particular pointed to the great importance of new leisure experiences (B_III_REF_033); they sometimes valued women-only offerings (C_VI_REF_085, D_VIII_REF_122), which were perceived as ‘safe places’. In one case, this even led to the independent organisation of a women-only meeting between German and refugee women (A_I_REF_012).

## 5 Discussion

Based on a salutogenic approach, which does not ask what makes people ill, but rather how they keep themselves healthy, this paper has first of all presented the self-reported health status of refugees, and secondly identified their health-promoting practices. Self-reported diseases in the settlement phase of interviewed refugees in rural Germany comprised both physical and psychological illnesses. Empirical data show that cardiovascular diseases and diabetes were reported, as well as musculoskeletal health impairments associated with having recently flown in overcrowded conditions, and psychosomatic diseases, all of which confirms the results of previous surveys (Goodman et al., 2018; Schröder et al., 2018). While health conditions were often explained as the result of experiences while travelling, the persistence, or even exacerbation of these diseases points to the fact that the structures of settlement and relatively short period of time since refugees’ arrival were not long enough to recover from diseases (cf. Silove et al., 2017). This emphasises the importance of post-medical understandings of health, which can be linked to a life course model, particularly in refugee research. Apart from this, the results show that perceptions of health status are not just subject-centred; nor do they include whole groups or societies, as often promoted by public health. Instead, as already suggested by Ryom et al. (2022), taking a household perspective offers the opportunity to identify the (health) constraints on family members both locally and at a distance, which may also influence individual health status. Finally, we identified the interdependence of health with other dimensions of integration (Ager and Strang, 2008). Good physical and mental health status enables inclusion and participation in the various realms of everyday life, including education, employment and housing. Conversely, the persistence of existing health-related constraints or the development of new conditions can impede access to society and thus hinder inclusion (Hecker and Neuner, 2019).

Regarding experiences of health care infrastructure and actors, general practitioners (GPs) could be identified as crucial and personally known. The study thus ties in with discussions surrounding the ethics of care in the relationship between doctors and patients in rural areas, although these discussions have not yet been linked to migration (Quilliam et al., 2023). While the existence and ease of access to GPs was perceived positively, specialists were seen to be hard to access. Moreover, various exclusionary practices during consultations were reported: culturally-induced uncertainties in patient-doctor contact and therapies that patients perceived as undifferentiated resulted in misunderstandings. Even at the doctor’s, discrimination occurs. This highlights refugees’ need to consult doctors who speak the same language. The absence of culturally sensitive doctors and language mediators is especially challenging in rural areas, where there is less diversity than in bigger cities, shedding light on the importance of multi-dimensional concepts of accessibility (Penchansky and Thomas, 1981).

Refugees perceive leisure activities as a chance for distraction from an overwhelming life (Reis et al., 2020; Ryom et al., 2022), as an opportunity to enhance their physical and mental health and acquire health resources. At the same time, they see differences in leisure facilities, everyday rhythms and social norms between the rural place where they live and their country of origin, which may lead them to experiment with new leisure habits. In that sense, our findings confirm the existing evidence from studies conducted in Australia (Reis et al., 2020), Canada (Hurly, 2018) and Denmark (Ryom et al., 2022). However, given that rural areas have particular characteristics, the results also indicate that infrastructural conditions make it more difficult to access leisure facilities. Moreover, both time sovereignty and financial independence are far more important in rural areas, and a lack of either can make it almost impossible to get to leisure venues at a distance from their homes. Refugees also think of leisure as a way to gain and strengthen social contacts locally, which fosters individual agency and social well-being. The interviews revealed that refugees frequently walked or cycled in the natural environment, in parks and forests or by rivers or lakes. In contrast with empirical data on the physical inactivity of refugees in reception centres (Andersen et al., 2021; Schröder et al., 2018), these findings point to greater levels of activity in the settlement phase. The positive health effects of green and blue areas (e.g. Claßen and Bunz, 2018), as well as of walking (e.g. Hu et al., 2001; Williams and Thompson, 2013), have been referred to many times in the literature, even in relation to young people from refugee backgrounds (Sampson and Gifford, 2010). In that sense, rural places offer good conditions for strengthening refugees’ health resources.

## 6 Conclusion

From a salutogenic perspective (Antonovsky, 1996), this paper explored refugees’ self-reported health issues and their coping strategies. Leisure practices in the settlement phase, which has to date often been neglected in migration research, were also investigated. Finally, we considered the particular characteristics of rural areas, where access to health care infrastructure is assumed to be challenging and where the place itself offers health-promoting leisure assets.

Due to the specific configurations of health care infrastructure in rural areas, which are well-covered by GPs but not by specialists, the mitigation of health issues for refugees can be seen to be more challenging than in other spatial contexts. However, apart from spatial access, accessibility must be addressed in terms of diversity-sensitive facilities, including sensitivity to language and cultural differences. Moreover, our results showed an obvious persistence of mental ill health, something also indicated by previous studies (e.g. Fazel et al., 2005; Silove et al., 2017). Thus, a holistic understanding of health, as foreseen in the health-in-all policies approach, for instance, must be adapted more consistently. This study has identified health-related practices that go beyond acute treatment to encompass leisure practices, which are assumed to promote the physical and mental health of the individual. However, respondents did not make a direct connection between rehabilitation and prevention. Despite the fact that the results showed that refugees themselves are looking for new leisure activities and consider leisure to be an opportunity to acquire more room to manoeuvre in their lives in general, they often did not mention the improvement of their health as a goal. Besides, preventive health care is barely available in rural areas, especially in terms of mental health. Accordingly, follow-up studies should consider more closely the interrelation between various kinds of leisure practices and disease prevention, as well as being gender-sensitive.

How could places of prevention be operationalised? To put it in terms of commodification – and drawing on leisure studies – natural amenities or assets that characterise rural areas and green spaces are frequently associated with physical activity and health (Claßen and Bunz, 2018). Notions of nature and rural space, however, are culturally constructed to a certain extent and nature may also be understood as threatening. Thus, future research design should also address migrants’ perception of natural amenities and their functions, and tie into a discussion of therapeutic landscapes and the appropriation of places (Gesler, 1992).

To sum up, the study showed that a salutogenic approach to refugees’ health, which incorporates practices that support physical and mental health (i.e., leisure) as well as treatment and access to infrastructure, is crucial for a deeper understanding of their health status. In that sense, our results indicate that the persistence and dynamics of health problems can only be understood by means of a life-course perspective (Spallek et al., 2011) that considers the interrelations between experiences both before and during settlement. Finally, as the study showed, we also need to consider the various interdependencies between health and other realms of everyday life and spaces of integration, which are especially relevant in the settlement phase.

## Funding information

The research which led to this paper was supported by funds of the Federal Ministry of Food and Agriculture (BMEL) based on a decision of the Parliament of the Federal Republic of Germany via the Federal Office for Agriculture and Food (BLE) under the rural development programme (Funding code 2817LE036, project period 01.01.2018 – 31.05.2021).

## CRediT authorship contribution statement

**David Spenger:** Conceptualization, Formal analysis, Writing – original draft, Writing – review & editing. **Stefan Kordel:** Conceptualization, Data curation, Formal analysis, Investigation, Methodology, Project administration, Resources, Validation, Writing – review & editing. **Lukas Schorner:** Formal analysis.

## Declaration of competing interest

The authors declare that they have no known competing financial interests or personal relationships that could have appeared to influence the work reported in this paper.

## Footnotes

1 For reasons of transparency, all indirect and direct quotes presented here are tagged with a token that indicates Federal State (A-D), rural district (I-VIII) and interview number (REF_001-REF_139).
